# Role of Albuminuria in Detecting Cardio-Renal Risk and Outcome in Diabetic Subjects

**DOI:** 10.3390/diagnostics11020290

**Published:** 2021-02-12

**Authors:** Pia Clara Pafundi, Carlo Garofalo, Raffaele Galiero, Silvio Borrelli, Alfredo Caturano, Luca Rinaldi, Michele Provenzano, Teresa Salvatore, Luca De Nicola, Roberto Minutolo, Ferdinando Carlo Sasso

**Affiliations:** 1 Department of Advanced Medical and Surgical Sciences, University of Campania Luigi Vanvitelli, Piazza Luigi Miraglia 2, 80138 Naples, Italy; piaclara.pafundi@unicampania.it (P.C.P.); carlo.garofalo@unicampania.it (C.G.); raffaele.galiero@unicampania.it (R.G.); dott.silvioborrelli@gmail.com (S.B.); alfredo.caturano@unicampania.it (A.C.); luca.rinaldi@unicampania.it (L.R.); luca.denicola@unicampania.it (L.D.N.); 2 Renal Unit, Department of Health Sciences, “Magna Graecia” University, Viale Europa, 88100 Catanzaro, Italy; michiprov@hotmail.it; 3 Department of Precision Medicine, University of Campania Luigi Vanvitelli, Via De Crecchio 7, 80138 Naples, Italy; teresa.salvatore@unicampania.it

**Keywords:** type 2 diabetes, albuminuria, cardiovascular risk, renal outcome

## Abstract

The clinical significance of albuminuria in diabetic subjects and the impact of its reduction on the main cardiorenal outcomes by different drug classes are among the most interesting research focuses of recent years. Although nephrologists and cardiologists have been paying attention to the study of proteinuria for years, currently among diabetics, increased urine albumin excretion ascertains the highest cardio-renal risk. In fact, diabetes is a condition by itself associated with a high-risk of both micro/macrovascular complications. Moreover, proteinuria reduction in diabetic subjects by several treatments lowers both renal and cardiovascular disease progression. The 2019 joint ESC-EASD guidelines on diabetes, prediabetes and cardiovascular (CV) disease assign to proteinuria a crucial role in defining CV risk level in the diabetic patient. In fact, proteinuria by itself allows the diabetic patient to be staged at very high CV risk, thus affecting the choice of anti-hyperglycemic drug class. The purpose of this review is to present a clear update on the role of albuminuria as a cardio-renal risk marker, starting from pathophysiological mechanisms in support of this role. Besides this, we will show the prognostic value in observational studies, as well as randomized clinical trials (RCTs) demonstrating the potential improvement of cardio-renal outcomes in diabetic patients by reducing proteinuria.

## 1. Introduction

The clinical significance of albuminuria in diabetic subjects and the impact of its reduction on the main cardio-renal outcomes through the use of different drug classes represent one of the most interesting research focuses of the last years. The loss of glomerular selective filtration, the initial expression of renal damage and the consequent progression of chronic renal failure (CKD) have led to proteinuria being considered a complex marker of both nephropathy and cardiovascular (CV) risk [1,2,3]. In fact, the initial concept of glomerular damage was associated with the tubular damage caused by the pathological presence of proteins in the pre-urine, with consequent inflammatory activation of the tubule and alteration of the tubulo-glomerular feedback and electrolytes imbalance. On the other hand, albuminuria is associated with endothelial damage, which determines a high CV risk [4,5].

Therefore albuminuria, even at the lower concentrations represented by microalbuminuria (urine albumin/creatinine ratio—UACR—30–300 mg/g), is an important cardio-renal risk marker.

Although nephrologists and cardiologists have been paying attention to the study of proteinuria for years, type 2 diabetes mellitus (T2DM) is currently considered a condition in which increased urine albumin excretion ascertainsthe highest cardio-renal risk [6,7,8]. In fact, diabetes is a condition by itself associated, through various mechanisms [9,10,11], with a high risk of both micro- and macrovascular complications [12,13,14,15,16]. Moreover, proteinuria reduction in diabetic subjects by several treatments lowers the risk of both renal and cardiovascular disease progression [17].

Therefore, The 2019 joint ESC-EASD guidelines on diabetes, prediabetes and cardiovascular disease assign to proteinuria a crucial role in defining the level of CV risk in diabetic patients [18]. In fact, proteinuria is considered to target organ damage, and alone it is enough to stage the diabetic patient at very high CV risk, thus affecting the choice of anti-hyperglycemic drug class.

The purpose of this review is to present a clear update on the role of albuminuria as a cardio-renal risk marker, starting from pathophysiological mechanisms in support of this role. Besides this, we will show its prognostic value in observational studies and how randomized clinical trials (RCTs) have demonstrated the potential improvement of cardio-renal outcomes in diabetic patients by reducing proteinuria.

## 2. Pathophysiological Role of Albuminuria in Cardio-Renal Damage

Recently, several potential biomarkers seem to play a role in both the onset and progression of diabetic nephropathy (DN) (e.g., microRNAs, exosomes, long noncoding RNAs, and microparticles) [19]. These are accompanied by other well-known markers suggestive of different types of renal damage: glomerular (Transferrin, Type IV collagen, Cystatin C), tubular (liver-type fatty acid-binding protein “L-FABP”, neutrophil gelatinase-associated lipocalin “NGAL”, kidney injury molecule-1 ”KIM-I”, α1-microglobulin, fibroblast growth factor 23 “FGF 23”), fibrosis (transforming growth factor-β1 “TGF-β1”, connective tissue growth factor “CTGF”), oxidative stress (8-hydroxy-2′-deoxyguanosine “8-OHdG”) and inflammation (monocyte chemoattractant protein-1 “MCP-1”, IL-1, IL-6, IL-18, TNF-α). In particular, given the known crucial role of oxidative stress in the development of vascular damage in T2DM, it is not clear how its products could be included among markers of renal damage. Many studies have shown a potential self-sustainment of reactive oxygen species (ROS) on both inflammatory damage and cardiovascular risk. In fact, ROS scavenges nitric oxide (NO) and peroxy-nitrite, which are increased in diabetic patients, resulting in a reduced NO bioavailability. Among the other products of oxidative stress in T2DM, the advanced glycation end-product (AGEs) can stimulate the production of free radicals, which seems closely related to the endothelial damage found in patients with CKD [20,21]. However, albuminuria/proteinuria still remains the most widely used marker of DN in both clinical practice and clinical trials, likely because microalbuminuria from the 1980s has been described not only as a renal, though also as a CV risk factor in diabetic patients [22]. Moreover, almost at the same time, proteinuria has been reported as a relevant risk marker of CV mortality in the general population [23].

The absence of proteins, particularly of albumin, is an expression of both the integrity of the glomerular apparatus and the ability of the proximal tubule to absorb the modest quantities of albumin physiologically present in the glomerular filtrate. Therefore, a progressive increase in albuminuria excretion determines both glomerular damage due to the effect on mesangium and tubular damage due to excessive albumin absorption with inflammatory phenomena of the interstitium, resulting in an overall functional renal damage [24].

**Figure 1:**
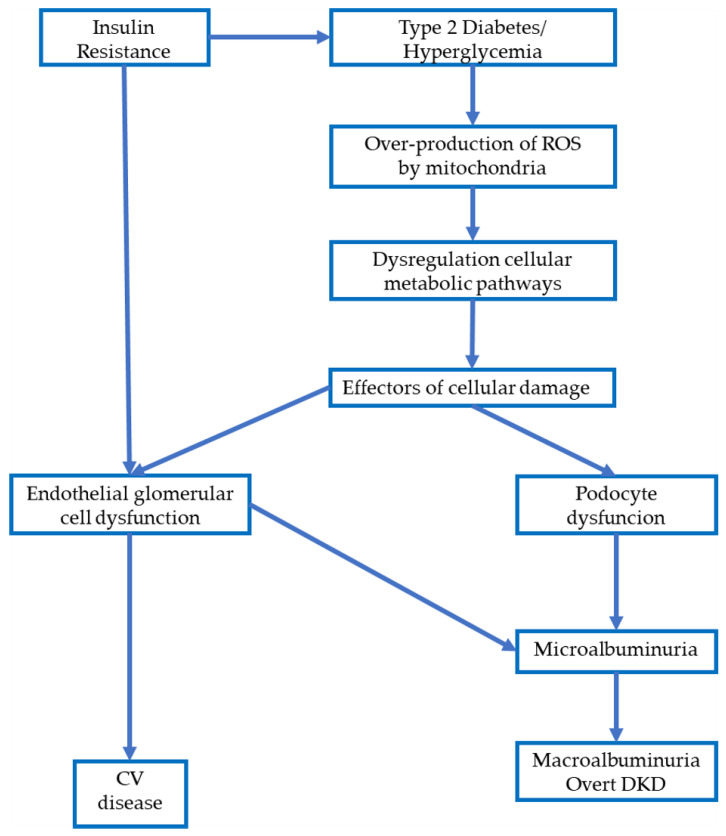
Pathophysiological cascade that describes the relationship between diabetes, endothelial damage, microalbuminuria, Diabetic Kidney Disease (DKD) and cardiovascular (CV) disease.

The link between renal and cardiovascular damage is explained by the Steno hypothesis, in which albumin escape is considered an expression of endothelial damage, with increased systemic vascular permeability [25,26]. Systemic vascular damage involves both glomerular and, subsequently, tubular damage, with consequent albuminuria and renal alteration, and at the same time, the endothelial damage causes increased CV risk (Figure 1).

Intriguingly, whether albuminuria is the cause or consequence of cardio-renal damage is currently an object of study. More recently, the hypothesis of a nonalbuminuric pattern in Diabetic Kidney Disease (DKD) has become much more challenging. Several studies, indeed, have shown that diabetic patients may develop a progressive end-stage chronic kidney disease regardless of albuminuria [27]. Moreover, as in the albuminuric pattern, these patients also seem more prone to developing CV events, particularly described as myocardial infarction and ictus, as compared to the general population [28]. Another interesting hypothesis, based on both experimental [29,30] and clinical data [31], is that variations in albumin excretion rate (AER) are constant with age and appear to have an individual character. The ability of the endothelium to regulate vascular tone through nitric oxide (NO) affects the predisposition to future renal damage [29,30]. Therefore, in “healthy” subjects, the different levels of albuminuria express different physiological conditions, as well as a predisposition to future renal and/or CV damage mediated by endothelial dysfunction [32]. Therefore, despite many other markers of glomerular and tubular damage that have been proposed, albuminuria still represents the main one suggestive at the same time, especially in diabetes mellitus, of both renal and cardiovascular damage.

## 3. Glomerular Mechanisms of Albuminuria

**Figure 2:**
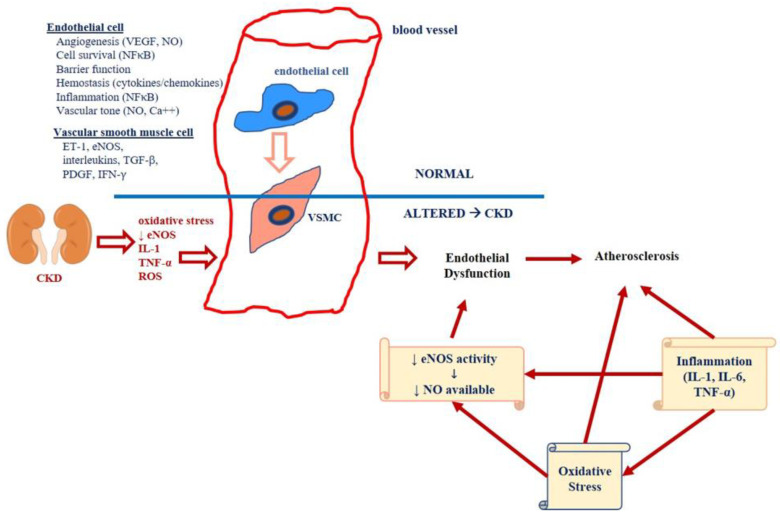
Effect of chronic kidney disease (CKD)-related factors on the vascular endothelium. Oxidative mediators involved in endothelial cell and vascular smooth muscle cell function and relationship with Chronic Kidney Disease (CKD). Abbreviations: eNOS, endothelial nitric oxide synthase; IL-, interleukin-; IL6, interleukin6; NF-κB, nuclear factor kappa-light-chain-enhancer of activated B cell; NO, nitric oxide; TNF, tumor necrosis factor-alpha; NO, nitric oxide; ET-1, endothelin 1; PDGF, platelet-derived growth factor; IFN-γ, interferon-gamma; TGF-β, tumor growth factor-beta; VEGF, vascular endothelial growth factor.

A central pathophysiological role in the onset and progression of micro- and subsequent macroalbuminuria/overt proteinuria is damage to the glomerular endothelium [33]. The impairment of the endothelial glycocalyx mediated by various mechanisms represents an early event of diabetic glomerular damage. Its effectors include vascular endothelial growth factor (VEGF), reactive oxygen species (ROS), inflammatory cytokines, endothelin-1 (ET-1), ET-1/endothelin receptor A (ETA) and Endothelial Nitric Oxide Synthase (eNOS). Similarly, there is a cross-talk between glomerular endothelial cells and mesangial cells, mainly mediated by platelet-derived growth factor-beta (PDGF-β)/PDGF-R [34] (Figure 2).

Moreover, all these factors and others released by the activated endothelium (e.g., ICAM-1, VCAM-1, vWF and matrix metalloproteinases) can induce an activation of the NF-κBintracellular pathway, thus determining an alteration of both intercellular cross-talk and cytoskeleton. This damage has been observed in several chronic diseases, such as CKD and heart failure [20]. The alteration of this cross-talk between endothelial cells and these other glomerular cells favors and increases endothelial damage. In fact, the increase in proteinuria is accompanied by morphological changes of the glomerulus, particularly lesion and loss of podocytes.

The podocyte injury in turn increases endothelial cell damage, thus creating a vicious cycle.

These morpho-functional changes are due both to the effects of hyperglycemia and the increased permeability of the glomerular filtration barrier to serum proteins [35].

This pathophysiological network confirms Steno’s hypothesis, indicating the alteration of the endothelial glycocalyx as the cause of both albuminuria and systemic endothelial dysfunction.

Glycocalyx dysfunction plays a central role in the development of vascular diseases, both micro- and macrovascular. Therefore, microalbuminuria is correctly considered a marker of generalized endothelial damage. Therefore, it can be used in clinical practice both as a predictor of the overall CV risk and, in the event of its reduction with therapeutic interventions, as an indicator of the reduction of CV risk [36], as well as of renal damage.

Recently, podocyte mitochondria have been reported to exert a key role in the development of proteinuria. Various types of damage can occur to podocytes, from hypertrophy to apoptosis, with a consequent reduction in the number of podocytes. This phenomenon contributes to the loss of the selective permeability of the glomerular membrane and consequent proteinuria.

Mitochondrial dysfunction of podocytes can be triggered by various mechanisms: the reduction of oxygen utilization at the mitochondrial level, ROS increase with consequent oxidative damage, advanced glycation end-products (AGEs) accumulation and endoplasmic reticulum stress. These mechanisms involve an alteration of intracellular homeostasis with consequent cell death. Several signaling pathways seem entangled in the genesis of damage to podocytes’ mitochondria and consequent proteinuria in Diabetic Kidney Disease (DKD), particularly the mammalian target of rapamycin (mTOR), Wnt/β-catenin and AMPK signaling pathways [37,38].

Furthermore, recently, a strict involvement of NLR family pyrin domain containing 3 (NLRP3) Inflammasome in several pathophysiological processes has been described, which leads to podocyte injury and plays a central role in albuminuria [39].

Therefore, both glomerular and cardiovascular damage can arise from a lesion of endothelial glycocalyx, mediated by various inflammatory effectors and triggered by hyperglycemia and elevated permeability. Once activated, this mechanism would portend to self-sustenance, and the consequent podocytes lesion would become responsible for the loss of proteins.

## 4. Tubular Mechanisms of Albuminuria

In recent years, in the pathogenesis of proteinuria, a strict interaction of the glomerulus and the proximal tubule in DKD has been described.

In experimental models of diabetes, a reduced reabsorption of albumin in the proximal tubules (PT) was observed as compared to control animals, which could partly be explained by a reduced albumin endocytosis by PT [40]. This “recovery hypothesis” has been confirmed more recently. Russo et al. have further elucidated the role of tubular reabsorption in the pathogenesis of albuminuria, observing that a large amount of albumin filtered from the glomerulus underwent a rapid recovery process by endocytosis from PT cells [41]. This finding was later confirmed in an experiment on rats rendered nephrotic by puromycin [42].

Interestingly, a role of the endocannabinoid system in the physiology of tubular epithelial cells has been described. In particular, in diabetes, we observe an increase in circulating endocannabinoids, with altered expression of their receptors in tubular epithelial cells and consequent cell damage ranging from hypertrophy to cellular dysfunction [43]. This involvement of endocannabinoids is very intriguing because their role in insulin resistance has been described [44].

Moreover, tubular involvement, especially in DKD, is worthy of utmost attention because, unlike tubular morphological changes, tubular injury is associated with the degree of renal dysfunction [45].

On the one hand, these hypotheses would confirm the close relationship between glomerular and tubular damage and, on the other, show the role of specific mediators for tubular damage.

## 5. Prognostic Role of Albuminuria on Cardio-Renal Risk

Abnormal albuminuria, defined as a value ≥ 30 mg/day, is an essential marker for staging chronic kidney disease (CKD) [46] and a recognized and powerful risk factor for adverse clinical outcomes in different settings, such as general population and patients with either T2DM, hypertension or CKD [47,48,49,50,51].

In the general population, the prevalence of abnormal albuminuria ranges between 4.8% and 10.3% [52], which significantly increases in high-risk populations such as patients with T2DM or hypertension.

The causative pathophysiological link between albuminuria and CV damage has been already described. Several studies have demonstrated a strong and linear association between albuminuria (even if moderately increased) and CV events in the general population [47,48], and in high-risk populations [53,54]. In addition, the community-based Framingham Heart Study (FHS) reported an association between the presence of abnormal albuminuria and a higher risk of incident heart failure (HF) (HR 1.71, 95% CI 1.25–2.34). In particular, albuminuria is associated with HF with reduced ejection fraction (HR 2.10, 95% CI 1.35–3.26) but not in the forms with preserved ejection fraction (HR 1.26, 95% CI 0.78–2.03) [55]. In the Multi-Ethnic Study of Atherosclerosis (MESA) study, diabetic subjects with albuminuria (micro- and macroalbuminuria combined) were 90% more likely to develop peripheral artery disease (1.90, 1.19–3.04) than those with no albuminuria [56]. For nondiabetic subjects, there were no statistically significant associations between albuminuria and vasculopathy [56]. However, the two studies, despite the large sample sizes, are either observational or cross-sectional, thus rendering it challenging to establish a causal effect of microalbuminuria and to generalize the results. Post-hoc analysis of the SAVOR-TIMI 53 trial in diabetic patients also identified an increased CV risk among patients with microalbuminuria, even if this was obtained just once, at the beginning of the trial [57]. In diabetic patients, even with a small increase in albuminuria (range 10–29 mg/g), the adjusted risk of cardiovascular death (aHR 1.65, 95% CI 1.24–2.21), ischemic stroke (aHR 1.43, 95% CI 1.02–2.01), myocardial infarction (aHR 1.73, 95% CI 1.33–2.24) and hospitalization for heart failure (aHR 1.65, 95% CI 1.22–2.21) was significantly higher [57]. Finally, in patients with overt diabetic nephropathy, the presence of albuminuria represents the most important factor predicting cardiovascular risk [54]. Patients with baseline albuminuria ≥ 3 g/g creatinine had a 92% higher risk (aHR 1.92, 95% CI 1.54–2.38) for the cardiovascular end point and a 2.70-fold (95% CI, 1.94 to 3.75) higher risk for HF compared with patients with low albuminuria (<1.5 g/g creatinine). Interestingly, modeling of the initial 6-month change in risk parameters showed that albuminuria reduction by 50% was the only predictor for cardiovascular outcome associated with an 18% and 27% reduction in CV risk and heart failure, respectively [58]. In a cohort of 742 T2DM patients with diabetic nephropathy, the risk of adverse CV events increased from 19% to 40% as estimated glomerular filtration rate (eGFR) declined from the CKD stage 1 to the stage 3b-5 and by 25% and 33% in micro- and macroalbuminuria, respectively [6]. Of note, this study reported a significant interaction between albuminuria and eGFR (*p* = 0.01), thus suggesting that albuminuria had a remarkable prognostic effect in subjects with high eGFR, virtually disappearing as eGFR became <30 mL/min/1.73 m^2^ [6].

Moreover, an increased albuminuria promotes higher tubular albumin reabsorption, with consequent intra-renal trafficking, which in turn activates the release of several inflammatory and pro-fibrotic mediators accelerating renal damage [59]. These mechanisms explain why albuminuria is now considered the principal risk factor predicting the faster progression of renal disease towards end-stage kidney disease (ESKD) [60,61,62,63,64].

Albuminuria acts as the main pathogenetic factor similarly in both diabetic and non-diabetic patients. Recently, a large multicentre observational study performed a direct comparison between CKD patients with the same proteinuria level either with or without T2DM [58]. Authors assessed the occurrence of different outcomes, including ESKD, in patients stratified by the presence of T2DM and proteinuria level (<0.15, 0.15–0.49, 0.5–1 and >1 g/day). They found that adjusted risk (aHR) for ESKD progressively increased across proteinuria strata and became significant with proteinuria in the range 0.5–1 (aHR 1.80 and 1.85, in diabetic and non-diabetic patients, respectively) and >1 g/day (aHR 2.70 and 2.69, in diabetic and non-diabetic patients, respectively), thus underlying a major and independent role of proteinuria [65]. However, although all patients received renin–angiotensin system (RAS) inhibitors at the maximally tolerated dose, no information of changes over time of proteinuria was available. Furthermore, albuminuria has an intrinsic pathophysiological limitation, being dependent not only on the extent of renal damage but also on the number and function of residual nephrons. Consequently, a low albuminuria level can herald a better prognosis in patients responding to antialbuminuric treatment or, alternatively, be merely a consequence of low eGFR. In the latter case, albuminuria alone may lose its prognostic significance likely because metabolic and hemodynamic factors associated with low eGFR play a major role in renal risk stratification. In this regard, the use of albuminuria (or proteinuria) indexed to eGFR as a more sensitive biomarker for the prediction of ESKD risk in a large cohort of CKD patients has been recently proposed [66]. This biomarker would allow an improvement in risk classification in more than 12% of patients, with higher reclassification in the elderly and in T2DM and advanced CKD, as well as in renal diseases characterized by higher proteinuria (diabetic nephropathy and glomerulonephritis) [66].

In recent years, there has been a growing interest in using change in albuminuria as a potential surrogate marker of CKD onset and progression rather than the single albuminuria level [60,67,68]. Sumida et al. reported, in a nationwide cohort of about 57,000 veterans (91% diabetics) with an eGFR ≥ 60 mL/min, a nearly linear association between 1-year changes in albuminuria and incident CKD. The aHR of incident CKD associated with mild albuminuria increase (1.25–2 times), and severe albuminuria increase (>2 times) were 1.12 (95% CI, 1.05–1.20) and 1.29 (95% CI, 1.21–1.38), respectively [68]. Moreover, Carrero et al. showed in a cohort of about 20,000 participants (61% diabetics) a strong association between change in albumin creatinine ratio (ACR) and the risk of ESKD [60]. The hazard of starting dialysis over a 2-year period was almost linear and did not differ according to diabetic status [60]. In contrast, several meta-analyses on data from clinical trials reported controversial results on the strength of associations between change in albuminuria and risk of ESKD [60,61]. However, their findings were weakened by the inclusion of highly selected populations, small sample sizes, and relatively short follow-up. Conversely, a recent individual participant-level meta-analysis of 28 observational studies including almost 700,000 subjects (80% with T2DM) provided robust results on the association between albuminuria change and subsequent risk of ESKD, with reliable estimates across a wide range of cohorts and subgroups [69,70,71]. This meta-analysis testified that a decrease of 30% in ACR during a baseline period of 2 years was associated with a 22% reduction in risk of ESKD, also accounting for regression dilution (HR 0.78, 95% CI 0.66–0.92). The lower risk of ESKD was consistent when albuminuria was quantified using UACR instead of albuminuria, when albuminuria changes were assessed at different time points (over 1 or 3 years) and when patients were stratified by the use of RAS inhibitors [71]. These analyses substantially improve the understanding of quantitative associations between the early change in albuminuria and the clinical endpoint of ESKD, and their application as a surrogate endpoint to groups of individuals in clinical trials. Mean change in albuminuria as an endpoint in clinical trials overcomes the inaccuracy of its estimation at the individual level, which results from substantial biological variation and laboratory measurement error. In particular, when corrected for regression dilution (regression to the mean effect), even modest changes in the true albuminuria level are reliably associated with meaningful changes in subsequent risk of ESKD. ADVANCE-ON, an extended 5 year follow-up of a diabetes clinical trial, is the only other study to adjust for regression dilution, obtaining similar findings in a population characterized by different ethnic groups [72]. All these results suggest, in the case of high baseline albuminuria, an association of a significant reduction in the risk of ESKD with even moderate true changes in albuminuria, rather than with imprecise single measure changes (e.g., 30% decrease).

## 6. Intervention Studies about the Reduction in Albuminuria and Risk of Kidney Outcome

Observational studies can only testify an association between albuminuria and ESKD but cannot prove a cause–effect relationship. The critical question of whether a drug-induced reduction in albuminuria value can predict renal protection may be answered only by examining randomized clinical trials (RCTs) and trial-based meta-analyses (Table 1).

Lambers-Heerspink et al. evaluated early albuminuria reduction in response to various pharmacological interventions as a predictor of the treatment effect on ESKD. The authors considered twenty-one RCTs, of which seven included only DKD patients [69]. As the main result, they observed a 24% ESKD risk reduction (95% CI, 11–34) for each 30% reduction in albuminuria. This association was consistent regardless of drug class (*p* = 0.73) and diabetic status (*p* = 0.89). However, the association between early changes in albuminuria and kidney events in this study is primarily based on trials of renin–angiotensin system blockade. Therapy with RAS inhibitors remains the main reno-protective intervention in DKD, although 30% to 50% of patients still present a high residual risk of ESKD [73]. More recently, a further meta-analysis of 41 RCTs with about 30,000 participants (71% with T2DM) has evaluated the relationship between treatment effects on albuminuria and renal endpoint by using a Bayesian mixed-effects meta-regression analysis [74]. Across all studies, therapeutic strategies reducing by at least 30% the geometric mean albuminuria as compared to control are associated with a 27% lower risk of composite renal endpoint (ESKD, eGFR < 15 mL/min/1·73 m^2^, or doubling of serum creatinine), with a meta-regression slope of 0.89 (95% Bayesian credible interval 0.13–1.70) [74]. The association was stronger in patients with baseline albuminuria > 30 mg/g but weaker for patients with low baseline levels of albuminuria [74].

In the last few years, the introduction in the therapeutic armamentarium of Sodium Glucose Transporter-2 inhibitors (SGLT2-i) has dramatically modified the renal risk of T2DM patients, mainly due to the hemodynamic and anti-albuminuric properties of these drugs. The beneficial renal effects of SGLT2-i were initially shown in the cardiovascular outcome trials EMPAREG, CANVAS and DECLARE, aimed at evaluating cardiovascular safety in T2DM patients, in which renal outcomes were assessed as secondary endpoints [75,76,77]. Besides these, the CREDENCE trial specifically aimed to evaluate the renal survival in a large population with T2DM with overt CKD receiving either Canagliflozin or placebo [78]. Overall, the results of these four large RCTs demonstrated, along with cardiovascular benefits [79,80], a major nephroprotective efficacy of SGLT2-i, with a significant 30% reduction in albuminuria and a 30–40% lower risk of progression to ESKD [66,67,68,69]. A recent post hoc analysis of the CREDENCE trial further investigated whether an early change in albuminuria after treatment with Canagliflozin is associated with long-term kidney outcomes [81]. Overall, Canagliflozin, as compared to placebo, reduced geometric mean ACR at 26 weeks by 31% (95% CI, 27% to 36%). Over a median follow-up of 2.2 years, 324 (8.4%) kidney outcomes were observed. This study demonstrated that each 30% decrease in albuminuria during the first months of treatment with Canagliflozin was associated with a 29% reduced risk of kidney outcomes in DKD patients (HR, 0.71; 95% CI, 0.67–0.76) [72]. Furthermore, despite early and sustained reductions in albuminuria with Canagliflozin, the authors observed residual albuminuria, which was associated with kidney and cardiovascular events both in placebo and Canagliflozin groups. This displays the importance of monitoring albuminuria during Canagliflozin treatment to better target both renal and CV prognosis.

The CREDENCE demonstrated a stronger association between change in albuminuria and kidney outcomes rather than for CV outcomes, underlying the central role of albuminuria as a risk factor for kidney events, whereas CV risk is determined by multiple other factors, including hyperglycemia and hyperlipidemia. The reduction of the renal endpoint was larger for higher baseline levels of albuminuria. Nevertheless, the reductions in albuminuria might explain around 50% of the treatment effect on the primary kidney outcome. This finding is consistent with recent data from the CANVAS Program [82]. However, it should be kept in mind that Canagliflozin, as well as the other SGLT2i, cannot be used in patients either with a low eGFR or in dialysis. Hence, the effects on these patients are unknown. In addition, as pointed out by the authors, there are few albuminuric patients. On the other hand, indeed, a beneficial effect of gliflozins also in normoalbuminuric patients should be mentioned, which suggests the involvement of other drug-related mechanisms in kidney protection.

Renal-protective effects associated with a significant reduction in albuminuria values were further observed with another class of drugs, selective antagonists of endothelin A receptor (ETA-RA). In particular, the SONAR trial found an ACR reduction from baseline of 51.8% (95% CI, 51.4–52.4) in about 2700 albuminuric T2DM patients with reduced eGFR responding to Atrasentan in the enrichment period [83]. This reduction was further associated with a lower risk of primary composite renal endpoint (sustained doubling of serum creatinine, eGFR < 15 mL/min/1.73 m^2^, chronic dialysis, kidney transplantation, or death from kidney failure) in subjects treated with Atrasentan (HR: 0.65 (95% CI 0.49–0.88)) after a median follow-up of 2.2 years [83]. A more recent post hoc analysis of the SONAR trial was focused on a small subgroup (n = 14) of patients treated with a combination of SGLT2-i and Atrasentan during the 6-week active open-label (enrichment) period [84]. These 14 patients were matched (1:3 ratio) with patients under Atrasentan monotherapy. Dual administration of SGLT2-i and Atrasentan was associated with a 28% (95% CI, 4–46%) larger reduction in ACR as compared to Atrasentan alone, hence suggesting a beneficial synergistic effect from the combined therapy resulting in a higher albuminuria reduction and renoprotection. These promising findings support future RCTs designed to assess the long-term efficacy and safety of a combined therapy with ETA-RA and SGLT2-i in high-risk patients with T2DM and CKD [84].

Finally, new interesting insights in the renoprotective strategies arise from the Finerenone in Reducing Kidney Failure and Disease Progression in Diabetic Kidney Disease (FIDELIO-DKD) trial [85]. This study was designed to test the hypothesis that Finerenone, a non-steroidal selective mineralocorticoid receptor antagonist, slows the progression of kidney disease and reduces both CV morbidity and mortality among DKD patients. As compared to placebo, Finerenone was associated with a 31% greater reduction of ACR during the first 4 months of the trial, which persisted in the following 30 months. More importantly, Finerenone also reduced the incidence of the primary composite kidney outcome (defined as kidney failure, sustained decrease of at least 40% in the eGFR from baseline or death from renal causes) by 18% (HR: 0.82; 95% CI, 0.73–0.93). Notably, the renoprotective effect of Finerenone was mainly driven by the sustained eGFR decline ≥ 40% (HR 0.81 (95% CI 0.72–0.92)), which is a validated surrogate endpoint of renal progression [86]. Hyperkalemia-related adverse events occurred more frequently with Finerenone than with placebo (18% and 9.0%, respectively), which was expected, also considering that >50% of T2DM enrolled patients had eGFR < 45 mL/min/1.73 m^2^. However, the discontinuation rate due to hyperkalaemia was only 2.3%. The benefit with respect to CKD progression was lower than that reported with Canagliflozin in the CREDENCE trial, more likely because SGLT2-i were allowed in the FIDELIO-DKD, thus leading to a higher renoprotection in the placebo group, whereas patients treated with mineralocorticoid receptor antagonists were excluded from the CREDENCE trial [78].

In conclusion, all the renoprotective new drugs currently available act producing an early and sustained reduction in albuminuria. This effect translates in the long-term in a significant reduction in renal events. Change in albuminuria is a biologically reasonable surrogate endpoint for the progression of CKD in RCTs, and clinicians must consider the early decrease in albuminuria as a reliable biomarker of a favorable response to treatment. Whether SGLT2-1, ETA-RA and mineralocorticoid receptor antagonists exert a significant impact in reducing renal events and also in the non-albuminuric phenotype of T2DM patients still remains to be verified by dedicated studies.

## 7. Non-Pharmacological Approach for Proteinuria Reduction

Besides pharmacological treatment, some non-pharmacological strategies have been proposed to reduce albuminuria in both diabetic and non-diabetic patients with CKD. The most common approaches include reduction of protein intake and low sodium diet [93]. Experimental models have demonstrated a large efficacy of a low protein diet (LPD) in reducing glomerular hyperfiltration by inducing vasoconstriction of afferent arterioles, with a subsequent decrease in the intraglomerular pressure. In this way, LDP pre-glomerular effects can act synergistically with the post-glomerular effects of RAS-inhibitors (efferent vasodilation). In addition, improvement in glomerular hyperfiltration may reduce renal inflammation by limiting the release of both pro-inflammatory and pro-fibrotic cytokines from the mesangial cells [94]. A very recent meta-analysis has reported a significant reduction of proteinuria and a slower decline over time of eGFR in DKD patients following a daily protein intake limited to less than 0.8 g/kg/day [95]. These data were consistent with other meta-analyses, although these latter included a lower number of studies and subjects [87,92,96]. LPD benefits may also include improvements in lipid and glucose control, especially in subjects at an early stage of kidney disease [95]. Among low-protein diets for CKD, there is a very restrictive one, which is supplemented with aminoacids and ketoacids (LPD-KA) in order to cover the minimum body nitrogen need and to minimize the nitrogen load. The LPD-KA diet has been proven to achieve a more effective metabolic control, a reduction of CV risk factors and also a slow-down of CKD progression by delaying the need for dialysis [88,89]. On the other hand, a very restricted renal diet may expose the patient with CKD to a higher risk of insufficient nutrient intake and protein-energy wasting, particularly in diabetic patients, in which the higher degree of inflammation, insulin resistance and more frequent hyper-catabolism might require more proteins and aminoacids to compensate the catabolic status. In a recent observational study, Bellizzi et al. reassured clinicians on the long-term effects of LPD-KA in diabetic CKD patients by testifying that a low-protein diet supplemented with ketoacids improves the accumulation of uremic products and ameliorates both glucose control and insulin sensitivity [90]. This dietary strategy did not worsen the nutritional status while preserving the body composition in diabetic patients with CKD.

Despite the encouraging results and the evident pathophysiological link between protein intake and proteinuria, further randomized trials are needed to confirm the usefulness of LPD as renoprotective strategy. Indeed, the most recent nutritional guidelines indicate as reasonable the prescription of a dietary protein intake between 0.6 and 0.8 g/kg/day in diabetic kidney disease patients to maintain a stable nutritional status and optimize glycemic control [91]. Despite this statement only being an opinion, being supported by too low-quality evidence to produce a graded recommendation, it is of utmost importance to provide some guidance both to patients and practitioners.

Notably, LPD can be synergistic with the direct effect of a low-sodium diet [93]. In all adults with CKD [87,95,96], it is recommended to limit sodium intake < 100 mmol/day to reduce blood pressure and proteinuria [91]. In the short term both observational and randomized studies, sodium restriction is effective in correcting the volume overload occurring since the early stages of CKD and, consequently, reduces blood pressure (BP) and proteinuria [97,98,99,100]. Similarly, in a pooled analysis of IDNT and RENAAL trials in patients with diabetic nephropathy, the beneficial effect of RAS inhibitors on both renal and cardiovascular outcomes is higher in patients following a low-salt diet (LSD) [101]. Recently, in a meta-analysis of 11 RCTs including 738 CKD patients (Stage 1–4, 46% with T2DM), LSD was associated with a significant decline of clinical and 24 h outpatient BP, as well as of proteinuria (−0.4 g/day, 95% CI from −0.55 to −0.22, *p* < 0.001) [102]. Changes in proteinuria display a linear correlation with changes in systolic BP, suggesting that the anti-proteinuric effect of sodium restriction may be dependent on BP reduction. On the other hand, it cannot be ruled out that LSD may exert its anti-proteinuric effect by enhancing the efficacy of drugs inhibiting RAS. Indeed, in a small randomized cross-over trial on 52 non-diabetic CKD patients, LSD enhanced the anti-proteinuric effect of lisinopril by obtaining a proteinuria reduction (51%) even higher than that detected after dual RAS blockade (21% after lisinopril and valsartan combination) [98]. When considering that both CKD and diabetes are characterized by a “salt-sensitive” phenotype, one would expect that favorable effects obtained in non-diabetic patients might be extended to the entire diabetic population.

## 8. Proteinuria Reduction and Cardiovascular Prevention in T2DM: RCTs-Based Evidence

Significant evidence has shown how altered levels of proteinuria and micro/macroalbuminuria are suggestive not only of renal damage, but also of cardiovascular diseases [6]. In addition, they may also represent a negative prognostic factor for the worsening of both renal and cardiac disorders [65]. For this reason, several trials related to both anti-proteinuric and anti-hyperglycemic drugs have assessed whether proteinuria reduction could positively affect cardiovascular outcomes (Figure 2).

Before the marketing of the new anti-hyperglycemic drugs—Dipeptidyl peptidase 4 inhibitors (DPP-4i), Sodium Glucose Cotransporter2 inhibitors (SGLT2i) and glucagon-like peptide 1 (GLP1ra)—the only drugs reported as effective towards high levels of proteinuria, with subsequent renal and cardiovascular protection, were Angiotensin-converting enzyme inhibitors (ACE-i) and angiotensin receptor blockers (ARBs).

MICRO-HOPE findings are very promising. The authors have assessed both renal and cardiovascular outcomes in a subpopulation from the HOPE trial, characterized by type 2 diabetes and microalbuminuria. ACEi has been reported to reduce both single and composite cardiovascular outcomes, with a subsequent reduction of up to the 16% of renal damage progression [103]. Conversely, in the DIABHYCAR study, a low dosage of Ramipril (1.25 mg/die) in about 5000 T2DM patients with persistent proteinuria did not prove effective in reducing the composite cardiovascular outcome despite a remarkable improvement of renal markers [104]. These differences in results could depend on the different study designs of these trials in terms of enrollment criteria and ACEi dosage. This latter point could suggest a dose-dependent effect of the ACEi, with a maximal benefit from higher dosages.

Likewise, ARBs have demonstrated a protective role in both renal and cardiovascular protection. Most studies have particularly focused on cardiovascular outcomes in T2DM patients with kidney dysfunction. The RENAAL study, which involved about 5000 patients with diabetes and nephropathy, reported losartan’s effectiveness on cardiovascular outcomes by reducing microalbuminuria [58]. In fact, the authors found that microalbuminuria was the main marker of cardiovascular damage, particularly in the presence of an anti-proteinuric effect > 30%. However, the physiopathological mechanisms are still unclear.

In contrast to the previous studies, losartan, despite reducing proteinuria, does not seem to exert a protective CV effect [105]. In particular, a recent study reported that both monotherapy and ACE/ARBs combination do not increase renal and cardiovascular efficacy [106]. As reported by other authors, indeed, ACEi effectiveness on cardiovascular protection might be due to a decrease of lipoproteins, with a consequent reduction of the atheromatic plaque [107]. However, this latter seems characterized by several limitations, particularly a low sample size and the absence of a placebo control group. As such, the authors suggested the need for large-scale trials and studies with a longer follow-up to better assess this topic.

The relationship between either altered renal function or proteinuria/microalbuminuria and cardiovascular outcome in diabetic patients has also been assessed in relation to anti-hyperglycemic therapeutic regimens.

As expected, the post-hoc analysis of SAVOR-TIMI has shown a correlation between renal function/microalbuminuria and cardiovascular outcome [108]. However, Saxagliptin did not prove effective with respect to placebo, neither on the renal outcome nor on the cardiovascular, regardless of eGFR and kidney failure [57]. The TECOS study, which assessed sitagliptin efficacy on almost 15,000 patients, did not report a higher renal benefit as compared to placebo after a 4 year follow-up. However, the authors conducted the analyses stratifying only for eGFR, regardless of microalbuminuria and proteinuria assessment [109]. Alogliptin, indeed, up to now has never been tested for its relationship with renal function markers.

Among GLP-1ra, the LEADER study revealed a higher efficacy of Liraglutide with respect to placebo versus all MACEs and single cardiovascular outcomes in a population at high CV risk and eGFR < 60 mL/min, regardless of baseline microalbuminuria [110].

In the SUSTAIN 1–7, which assessed the effects of Semaglutide as compared to both placebo and other competitors, the drug showed significant protection on the cardiovascular outcome. In SUSTAIN 1–5 and 7, this effect was accompanied by an overall reduction in eGFR, whilst in SUSTAIN 6, no difference was observed. In addition, the albuminuria-lowering effects were higher in patients with either microalbuminuria or macro albuminuria in the semaglutide or active-comparator treatment groups, while no effect was observed in the normo-albuminuria group [111]. These findings are likely limited by a short follow-up.

More recently, the REWIND study, which compared Dulaglutide with placebo in patients at high cardiovascular risk, reported a reduction at 5-years of the risk of macroalbuminuria development of 23% (HR 0.77, 95% CI 0.68–0.87; *p* < 0.0001). The authors suggest that this behavior, in relation to the findings, may also be extended to the cardiovascular outcome, due to the effects of the drug on both blood pressure and endothelial damage [112].

Among SGLT2i, a post-hoc analysis from the EMPAREG-Outcome reported a 18% reduction in urinary albumin creatinine ratio (UACR) with Empagliflozin as compared to placebo and an increased likelihood of a UACR decrease > 30%. This reduction in the short term also was significantly associated with a lower risk of both cardiovascular and renal outcomes in the long term [113].

Consistently with the EMPAREG Outcome findings, in the CANVAS clinical trial, Canagliflozin displayed significant non-inferiority and superiority of the composite cardiovascular outcome (death from cardiovascular causes, nonfatal myocardial infarction, or nonfatal stroke) with a risk reduction (RR) of 14% (HR 0.86, 95% CI 0.75–0.97; *p* < 0.001 and *p* = 0.002, respectively) compared to placebo, although no difference was observed on the single outcomes. In parallel, Canagliflozin also positively affected both the progression of albuminuria (HR 0.73, 95% CI 0.67–0.79) in those with a baseline normo/micro/macroalbuminuria profile and its regression (HR 1.70, 95% CI 1.51 to 1.91) [114]. This topic has been further assessed in the DECLARE-TIMI 58. In particular, the authors observed, in a population of about 17,000 patients, non-inferiority of Dapagliflozin as compared to placebo regarding the risk of MACE, despite a non-significant rate reduction of MACE. However, the significantly lower rate of CV death and hospitalization for HF in different sub-groups allows us to suppose an overall cardiovascular benefit [115]. Consistently with the CV outcome, DECLARE authors, as already previously reported by Petrykiv et al., further reported an improvement of renal function (in terms of eGFR and microalbuminuria reduction) associated with a concomitant improvement of CV outcome [116].

In conclusion, ACEi and ARBs largely demonstrated an anti-proteinuric and cardioprotective effect. Beyond them, in recent years, among anti-hyperglycemic drugs, GLP-1ra and SGLT2i have proven effective in reducing either the progression or the regression of microalbuminuria, thus resulting in a cardiovascular benefit also in patients with overt renal damage.

## 9. Conclusions

Albuminuria is a marker of renal damage and CV risk in diabetic subjects. Currently, albuminuria/proteinuria still represents an important prognostic factor for the onset and progression of both DKD and CVD. Several studies have demonstrated a beneficial effect of its reduction in both kidney and heart as exerted by new drugs. However, new RCTs are needed to confirm these exciting findings, especially with a longer follow-up.

## Figures and Tables

**Table 1:** Findings from the randomized clinical trials on cardiorenal outcome with antihypertensive and anti-hyperglycaemic drugs.

RCT	Publication Date	Active Drug/ Comparator	No. Patients	Median Follow-Up (yrs.)	Baseline eGFR (mL/min/m^2^)	Baseline Albuminuria	Risk of Composite CV Endpoint	Risk of CV Death	Risk of Composite Renal Outcome	Reduced Proteinuria
**HOPE/MICRO-HOPE [83]**	2000	Ramipril vs. Placebo	3577	4.5	n.a.	553 mg/g	−25%	−37%	−16%	9%
**RENAAL [59,85]**	2001	Losartan vs. Placebo	1513	3.4	39.5	1168 mg/g	−10%	−2%	−16%	−35%
**DIABHYCAR [84]**	2004	Ramipril vs. Placebo	4912	4	n.a.	65 mg/L	−3%	3%	−7%	−19%
**SUSTAIN-6 [87]**	2016	Semaglutide vs. Placebo	3297	2.1	76.1	38.6 mg/g	−26%	−2%	n.a.	n.a.
**EMPA-REG [71,88]**	2017	Empagliflozin vs. Placebo	7020	3.1	74.2	25.51 mg/L	−14%	−38%	−46%	n.a.
**CANVAS [72,78,89]**	2017	Canagliflozin vs. Placebo	10,142	2.4	76.7	12.3 mg/g	−14%	−13%	−40%	−27%
**ADVANCE-ON [68]**	2018	Perindopril/Indapamide vs. Placebo	8766	7.7	n.a.	48.7 mg/g	−16%	−19%	−37%	n.a.
**CREDENCE [74,78]**	2019	Canagliflozin vs. Placebo	4401	2.62	56.3	923 mg/g	−20%	−22%	−30%	−40%
**DECLARE-TIMI58 [73,90]**	2019	Dapagliflozin vs. Placebo	17,160	4.2	85.4	n.a.	−7%	−2%	−47%	n.a.
**DAPA-HF [91]**	2019	Dapagliflozin vs. Placebo	4744	1.52	66	n.a.	n.a.	−18%	−29%	n.a.
**REWIND [92]**	2019	Dulaglutide vs. Placebo	9901	5.4	77.2	15.93 mg/g	−12%	−%	−15%	−23%
**SONAR [79,80]**	2019	Atrasentan vs. Placebo	11,087	2.2	44	797 mg/g	−12%	10%	−35%	39%
**FIDELIO-DKD [81]**	2020	Finerenone vs. Placebo	5734	2.6	44.4	833 mg/g	−14%	−14%	−18%	−19%
